# Anticoagulation management in a pediatric patient with protein S deficiency and acute pulmonary embolism undergoing ECMO-supported intervention: a case report

**DOI:** 10.3389/fped.2026.1956558

**Published:** 2026-10-22

**Authors:** Chen Geng, Miao Zhang

**Affiliations:** Department of Intensive Care Medicine, Qilu Hospital (Qingdao), Cheeloo College of Medicine, Shandong University, Qingdao, Shandong, China

**Keywords:** anticoagulation management, case report, extracorporeal membrane oxygenation, pediatric nursing, protein S deficiency, pulmonary embolism, thrombophilia

## Abstract

**Introduction:**

Protein S deficiency is a predominant form of hereditary thrombophilia in Asian populations. Pediatric venous thromboembolism (VTE) is exceedingly rare, with an incidence of only 0.2%–0.6%, and acute massive pulmonary embolism (PE) requiring extracorporeal membrane oxygenation (ECMO) support in children with thrombophilia presents exceptional clinical challenges. The core therapeutic dilemma lies in reconciling the hypercoagulable state inherent to protein S deficiency with the high bleeding risk following thrombolytic therapy, compounded by the obligatory anticoagulation requirement during ECMO. This case report describes the nursing management of a pediatric patient with protein S deficiency and acute PE who successfully underwent ECMO-supported interventional treatment under high bleeding risk.

**Outcome:**

The patient was successfully weaned from ECMO after 98 h of support, extubated on day 6, and discharged in stable condition after 30 days of hospitalization. At 6-month follow-up, no thrombus recurrence was observed, and the patient had fully resumed normal life and academic activities.

**Conclusion:**

This case demonstrates that an individualized low-dose heparin anticoagulation strategy, guided by the PEACE consensus and supported by meticulous monitoring, can effectively balance the competing risks of thrombosis and hemorrhage in pediatric ECMO patients with underlying thrombophilia and recent thrombolysis. Notably, as a single case report, this finding cannot be broadly generalized to all pediatric ECMO patients with thrombophilia. Instead, this case provides a potential framework for individualized anticoagulation and nursing management that requires further validation in larger pediatric cohorts. The nursing experience provides a practical reference for managing similar complex cases in pediatric critical care.

## 1 Introduction

Thrombophilia is a hypercoagulable state resulting from inherited or acquired deficiencies of anticoagulant proteins, coagulation factors, or fibrinolytic proteins, or from the presence of acquired risk factors (1, 2). Hereditary thrombophilia exhibits significant ethnic variation: coagulation factor abnormalities are more prevalent in Western populations, whereas anticoagulant protein deficiencies—including protein C deficiency and protein S deficiency—predominate in Chinese populations (1, 3). Pediatric venous thromboembolism (VTE) is exceedingly rare, with an incidence of only 0.2%–0.6%, of which pulmonary embolism (PE) accounts for approximately 15% (4, 5). Children with thrombophilia are at substantial risk of recurrent thrombotic events and poor outcomes. Cases of acute massive PE requiring ECMO-supported interventional therapy in children with thrombophilia are exceptionally rare, with few reports in the literature, and standardized nursing protocols remain largely unavailable. The management of such cases is particularly challenging due to the fundamental contradiction between the hypercoagulable state inherent to thrombophilia and the high bleeding risk following thrombolytic therapy, further complicated by the obligatory systemic anticoagulation required during ECMO. Studies have shown that up to 70.2% of pediatric ECMO patients experience bleeding complications, with 16% developing intracranial hemorrhage—bleeding is an independent risk factor for mortality in this population. The presence of prior intracranial hemorrhage, as in this case, further amplifies the bleeding risk and narrows the therapeutic window for anticoagulation (6).

This report describes a case of a 10-year-old girl with protein S deficiency and a history of cerebral venous sinus thrombosis with intracerebral hemorrhage who developed acute massive PE and was successfully managed with ECMO-supported interventional therapy under a carefully calibrated low-anticoagulation protocol. Notably, to the best of our knowledge, this is the first reported case of a pediatric patient with the unique combination of protein S deficiency, prior intracerebral hemorrhage, massive pulmonary embolism, systemic thrombolysis, and VA-ECMO support managed with such an individualized anticoagulation and nursing protocol. Existing literature has reported isolated cases of pediatric ECMO for PE, or pediatric thrombophilia management, but few have addressed the combination of these concurrent high-risk factors, which creates an extreme therapeutic dilemma. Furthermore, our nursing management protocol, which integrates dynamic bleeding risk surveillance, closed-loop anticoagulation titration, and family-centered support, differs from previously reported cases by providing a more systematic, risk-stratified approach that balances competing risks more effectively. We detail the nursing management strategies that enabled the successful navigation of this therapeutic dilemma, with the aim of providing a practical reference for clinicians and nurses managing similar rare and complex pediatric cases.

## 2 Case presentation

### 2.1 Patient information

A 10-year-old female was admitted to our vascular surgery department on September 17, 2024 (Day 0 of admission, with a 1-day history of sudden swelling and pain in the left lower extremity. Ultrasound examination revealed thrombosis involving the bilateral iliac veins and the left common femoral vein, as well as thrombosis at the orifice of the left great saphenous vein.

Past medical history: 11 months prior to admission, the patient was treated at an outside hospital for cerebral venous sinus thrombosis complicated by cerebral infarction and intracerebral hemorrhage. During that admission, laboratory investigations revealed elevated lupus anticoagulant, antithrombin III 68.5%, protein C 116.9%, and protein S 55.2% (reference range: 60%–130%). Genetic testing for F5/F2 gene variants associated with venous thrombosis risk was negative. A diagnosis of protein S deficiency-associated thrombophilia was established. No significant family history of thrombosis or bleeding disorders was reported. The patient was discharged on rivaroxaban 10 mg once daily but discontinued the medication voluntarily in March 2024.

### 2.2 Diagnostic assessment and clinical course

On Day 4 of admission, in the morning, the patient presented with sudden chest pain, severe dyspnea, and rapidly deteriorating hemodynamics (heart rate increased to 142 bpm, blood pressure dropped to 85/50 mmHg, SpO2 decreased to 82% on room air). Given her known history of deep vein thrombosis (DVT) and thrombophilia, we immediately suspected acute massive PE. In the early afternoon, emergency endotracheal intubation was performed, and mechanical ventilation was initiated. Intravenous alteplase thrombolysis was administered. The patient was transferred to the Pediatric Intensive Care Unit (PICU).

Upon PICU admission, the patient's oxygenation index was 41.0 on 100% FiO₂. Echocardiography demonstrated right ventricular enlargement, reduced right ventricular systolic function, and pulmonary hypertension. The chest x-ray showed bilateral patchy opacities in the lower lung fields, with an enlarged cardiac silhouette, consistent with acute pulmonary edema and right heart strain secondary to massive PE. This chest radiograph is presented in Figure 1. Inhaled nitric oxide was administered to reduce pulmonary artery pressure. Given the combined need for adequate oxygenation and additional cardiac support to stabilize the patient's hemodynamics and respiratory function, VA-ECMO was emergently established later that afternoon. Meropenem and vancomycin were initiated for empiric antimicrobial coverage

**Figure 1:**
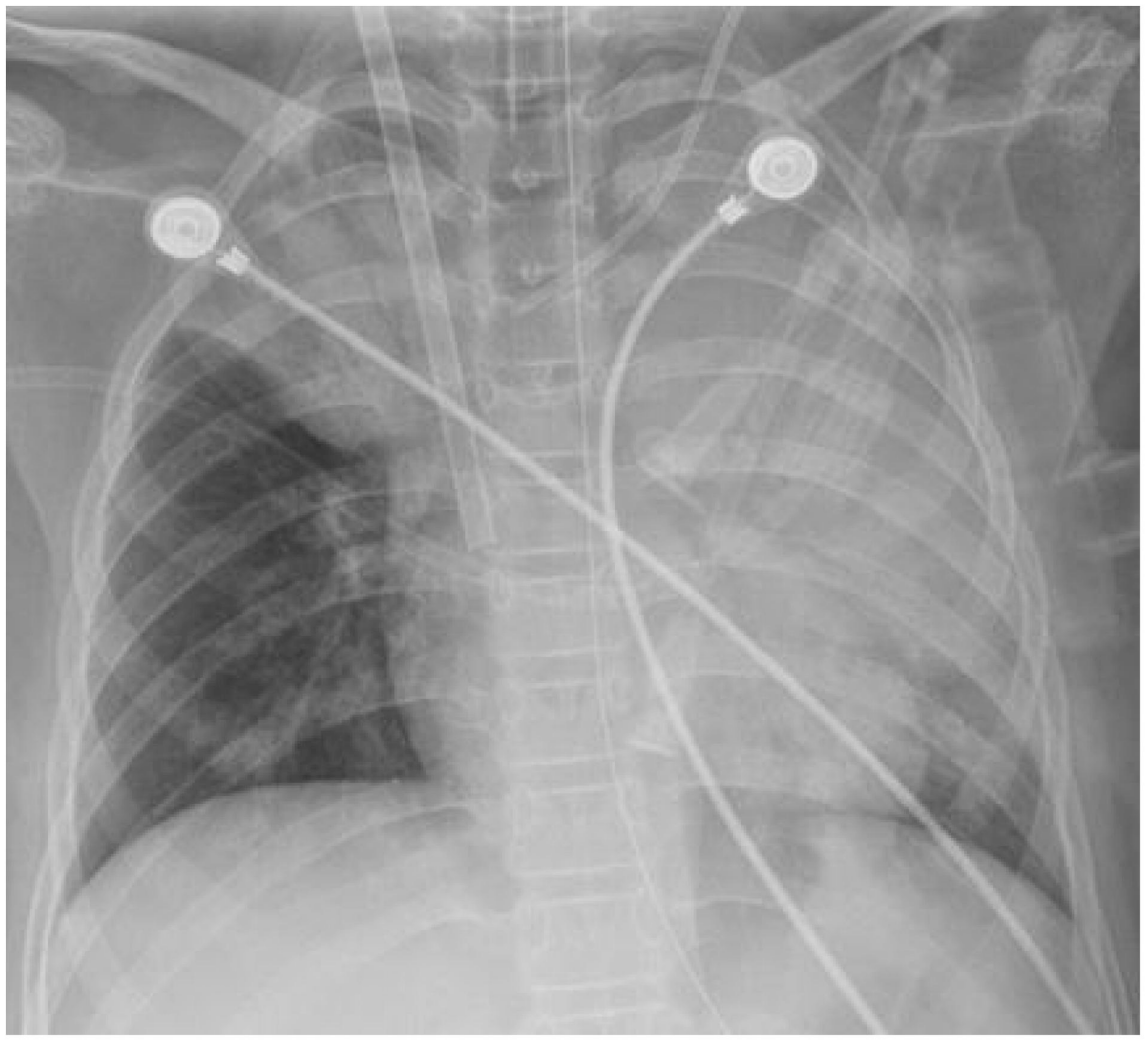
The chest x-ray results on September 21 (day 4 of admission).

7 h after the systemic alteplase administration, under ECMO support, the patient underwent pulmonary artery thrombus fragmentation, thrombolysis, and aspiration in the interventional suite. The rationale for combining these techniques was that systemic thrombolysis alone had not achieved sufficient recanalization, and combining mechanical thrombus fragmentation, aspiration, and local low-dose thrombolysis could achieve more effective recanalization while minimizing the dose of thrombolytic agent, thus reducing the systemic bleeding risk. Angiography revealed main right pulmonary artery occlusion and partial left lower pulmonary artery embolism. Following repeated mechanical fragmentation and aspiration, residual organized thrombus remained, so a multi-side-hole thrombolytic catheter was placed for local continuous thrombolytic infusion with alteplase at 0.5 mg/kg/h for 12 h.

On Day 5 of admission, the patient's left-ventricular ejection fraction (LVEF) declined to 35%, accompanied by left-ventricular wall-motion abnormalities, indicating secondary left-ventricular dysfunction resulting from severe systemic hypoperfusion and myocardial stunning. Intra-aortic balloon pump (IABP) support was therefore initiated. Following 24 h of IABP support and optimized pharmacotherapy, the patient's LVEF recovered to 65%, and IABP support was subsequently discontinued. On Day 8 of admission, a standardized ECMO-weaning trial was performed, in which ECMO flow was gradually reduced to one-third of the baseline flow over 4 h. During the trial period, the patient maintained stable hemodynamics with satisfactory oxygenation (PaO₂/FiO₂ > 300) and normal lactate levels. Repeat echocardiography confirmed normalized right ventricular function and a left-ventricular ejection fraction (LVEF) of 65%, demonstrating readiness for ECMO decannulation. The ECMO circuit was removed after 98 h of support. Figure 2 shows the chest-CT images. The post-intervention CT confirmed significant resolution of the pulmonary embolism. On Day 10 of admission, extubation readiness was confirmed based on adequate spontaneous breathing effort, intact airway-protective reflexes, stable hemodynamics, and a negative cuff-leak test. The endotracheal tube was subsequently removed, and oral feeding was resumed.

**Figure 2:**
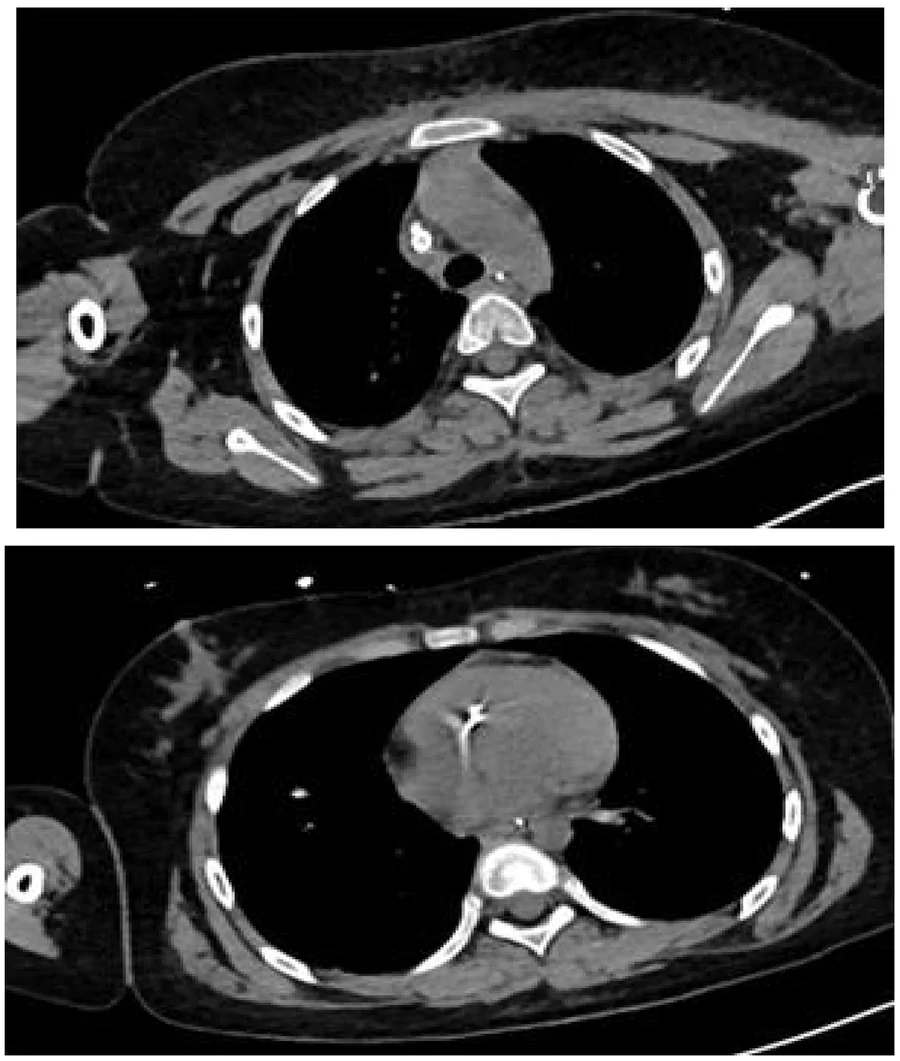
The chest CT images results on September 25 (day 8 of admission).

The patient was discharged in stable condition on October 21, 2024 (On Day 34 of admission). After discharge, the patient was restarted on rivaroxaban 20 mg once daily for long-term anticoagulation, given her underlying protein S deficiency and history of recurrent venous thromboembolism. Recent studies have demonstrated the efficacy and safety of rivaroxaban in pediatric patients with VTE: Spiezia et al. showed that rivaroxaban has a favorable benefit-risk profile in pediatric VTE patients, with effective thrombosis prevention and low bleeding rates, supporting its use in this population (7). The patient continued this regimen during follow-up, and at 1-year follow-up, she had no recurrent thrombotic events or bleeding complications, and had fully resumed normal activities. The detailed timeline of key events during the patient's hospitalization and follow-up is summarized in Table 1.

**Table 1:** Timeline of key events.

Date/Time	Key Event
11 months prior to admission	Diagnosis of protein S deficiency-associated thrombophilia at outside hospital; rivaroxaban initiated
6 months prior to admission	Patient voluntarily discontinued rivaroxaban
Day 0: Admission with left lower extremity DVT	Admitted with left lower extremity DVT
Day 4, Morning: Acute massive PE onset	Acute massive PE following ambulation
Day 4, Early Afternoon: Endotracheal intubation, thrombolysis initiated	Endotracheal intubation, mechanical ventilation, intravenous alteplase thrombolysis
Day 4, Later Afternoon: VA-ECMO established	VA-ECMO established
Day 4, Evening: ECMO-supported pulmonary intervention	ECMO-supported pulmonary artery thrombectomy with multi-side-hole catheter placement
Day 5，Early Morning: IABP placement	IABP placement for left ventricular decompression
Day 6: IABP removal (LVEF 70%)	IABP removal (LVEF 70%)
Day 8: ECMO decannulation (98 h of support)	ECMO decannulation (98 h of support) and removal of thrombolytic catheter
Day 10: Extubation, hemostatic agents discontinued	Extubation, oral feeding resumed, hemostatic agents discontinued
Day 34: Discharge	Discharged
1year follow-up	Full recovery, no thrombus recurrence

## 3 Discussion

In this case, we successfully managed a case of acute pulmonary embolism (PE) in a pediatric patient with underlying Protein S deficiency, who presented with concurrent high-risk factors including prior intracerebral hemorrhage, systemic thrombolysis, and the need for extracorporeal membrane oxygenation (ECMO) support. This case highlights the exceptional clinical challenges posed by such rare and complex presentations, where the hypercoagulable state of thrombophilia must be carefully balanced against the high bleeding risk of thrombolytic therapy and mandatory ECMO anticoagulation. In this discussion, we detail the individualized management strategy we implemented to navigate this therapeutic dilemma, and contextualize our approach within the existing literature to provide a practical framework for future care of similar complex pediatric cases.

To support the patient's recovery, we implemented a comprehensive, tailored nursing framework that integrated multiple dimensions of care, including dynamic bleeding risk monitoring, closed-loop anticoagulation titration, supportive nutritional and infection control care, and family-centered psychological support. This systematic approach played a critical role in achieving the positive clinical outcome.

### 3.1 The anticoagulation dilemma: balancing thrombosis and hemorrhage

The central challenge in managing this case was the fundamental contradiction between the patient's hypercoagulable state—stemming from protein S deficiency—and the high bleeding risk following thrombolytic therapy, compounded by the systemic anticoagulation required for ECMO circuit maintenance. Protein S is a vitamin K-dependent anticoagulant protein that functions as a cofactor for activated protein C in the inactivation of factors Va and VIIIa (8). Deficiency of protein S results in a prothrombotic state, predisposing patients to recurrent VTE (9). In this patient, the protein S level of 55.2% confirmed the diagnosis.

However, the recent administration of alteplase for massive PE had induced a state of systemic fibrinolysis and coagulopathy, placing the patient at substantial risk for major hemorrhage—including recurrent intracranial hemorrhage given her prior history. The addition of ECMO, which requires continuous anticoagulation to prevent circuit thrombosis, created a precarious therapeutic equilibrium.

Traditionally, the approach to such high-bleeding-risk scenarios might have favored no anticoagulation or minimal anticoagulation. However, the 2025 Pediatric ECMO Anticoagulation CollaborativE (PEACE) consensus provided a critical evidence-based framework. For pediatric patients at high bleeding risk, the consensus recommends low-dose unfractionated heparin (UFH), targeting an ACT of 140–180 s (10). Building on this guidance, we implemented a more conservative and meticulously monitored protocol.

### 3.2 Individualized low-dose heparin protocol

Our anticoagulation strategy was characterized by three principles: low starting dose, slow titration, and monitoring. Initial dosing: UFH was initiated at 4 U/(kg/h) via a dedicated central venous line—substantially lower than the conventional ECMO anticoagulation range of 10–50 U/(kg/h) (11). This conservative starting point was chosen to minimize bleeding risk while providing baseline protection against circuit thrombosis.

Target ranges: Anticoagulation targets were set at ACT 140–160 s and APTT 50–60 s (11). These targets were deliberately lower than conventional ECMO targets (ACT 160–200 s, APTT 60–80 s) to accommodate the patient's heightened bleeding risk while still providing meaningful thromboprophylaxis.

Monitoring and titration: A closed-loop “monitor-assess-adjust” protocol was implemented. In addition to routine coagulation parameters (ACT, APTT), we also monitored platelet count, fibrinogen levels, and hemoglobin every day to comprehensively assess the anticoagulation status and bleeding risk. Coagulation parameters (ACT, APTT), platelet count, and fibrinogen levels were monitored every 4 h. If ACT fell below 140 s without evidence of active bleeding, the heparin infusion was increased by 0.5–1 U/(kg/h). If APTT exceeded 60 s or bleeding manifestations increased, the infusion was paused for 30 min and then resumed at a reduced rate. During the 98-h ECMO support, the heparin dose was adjusted only once: from the initial 4 U/(kg/h) to 5 U/(kg/h) on the first day after ECMO initiation, as the ACT dropped to 135 s without any bleeding signs, and was then maintained at 4.5–5 U/(kg/h) for the rest of the ECMO course. The platelet count decreased slightly from 225 × 10^9^/L at baseline to 122 × 10^9^/L at the lowest point, with no significant thrombocytopenia. Fibrinogen levels were consistently maintained between 2.0 g/L and 3.46 g/L, without significant hypofibrinogenemia. During the entire period, we performed multiple blood product transfusions given the patient's coagulation dysfunction and progressive anemia: we transfused a total of 9.5 units of packed red blood cells, 3,370 mL of fresh frozen plasma (including virus-inactivated plasma), and 40 units of cryoprecipitate, to correct the abnormal coagulation parameters and severe anemia, as the hemoglobin level dropped to as low as 56 g/L. Anticoagulation was adjusted dynamically according to the patient's coagulation and bleeding status, with temporary adjustments during the interventional and transfusion procedures. Minor oozing at puncture sites was observed, and we closely monitored the bleeding status as the patient was at high risk of bleeding. Regarding the choice of anticoagulant, we chose unfractionated heparin (UFH) rather than bivalirudin for this patient. At our center, UFH remains the first-line anticoagulant for pediatric ECMO, as we have extensive clinical experience with UFH monitoring and dose titration, and it has a well-characterized reversal agent (protamine sulfate) which is critical for patients at high bleeding risk, such as this patient with prior intracranial hemorrhage. While bivalirudin has advantages including predictable pharmacokinetics and reduced risk of heparin-induced thrombocytopenia (HIT), it lacks a specific reversal agent, which poses a significant risk in case of unexpected major bleeding. For this high-risk patient, the ability to rapidly reverse anticoagulation if needed was a key consideration, which led us to choose this individualized low-dose UFH protocol.

### 3.3 Multidimensional bleeding risk management

Beyond systemic anticoagulation, a comprehensive bleeding prevention strategy was essential. We implemented the following measures:

Standardized puncture site hemostasis: For ECMO cannulation sites (internal jugular vein and femoral artery), manual compression was applied for 30 min following any manipulation, followed by continuous local compression with a 1-kg saline bag for 24 h. This approach effectively controlled local oozing and prevented hematoma formation.

Systematic bleeding surveillance: A structured screening protocol was implemented: gastric occult blood testing every day; daily inspection of urine, stool, oral mucosa, and skin for evidence of bleeding; and hourly neurological assessments (pupillary response, consciousness level) for early detection of intracranial hemorrhage. A protocol was established for rapid response to hemoglobin drops >15 g/L within 4 h, triggering immediate transfusion of packed red blood cells and fresh frozen plasma.

When gastric occult blood testing became positive, we adjusted the enteral nutrition regimen, and administered acid suppression, gastric protection and hemostatic medications(iced-saline plus thrombini). This intervention successfully controlled gastrointestinal bleeding, with temporary adjustment of systemic anticoagulation during this period.

### 3.4 Supportive care

During the acute phase of treatment, we provided standardized supportive care to maintain the patient's clinical stability. This included tailored nutritional support adjusted to her gastrointestinal function and bleeding risk, as well as a checklist-based infection prevention bundle targeting common ECMO-associated complications. The patient remained free of catheter-related infections throughout hospitalization, and her nutritional status was well maintained without feeding intolerance or rebleeding.

### 3.5 Family-involved psychological support

Pediatric intensive care experiences can lead to lasting psychological sequelae, including post-traumatic stress disorder (PTSD), anxiety, and depression (12). We implemented a family-involved psychological support model:

Narrative nursing: After the patient regained consciousness, narrative therapy techniques were used to help the patient process her ICU experience. She was encouraged to describe her “scariest moments” and externalize her fears about the endotracheal tube and ECMO machine. Simple anatomical explanations and drawings helped restore her sense of safety.

Family empowerment: The mother was permitted to accompany the patient during designated daily visiting hours, with infection control precautions in place. Brief educational sessions were provided to help the mother recognize and respond to the patient's anxiety signals. The patient reported: “When Mom was with me and the nurse explained that the machines were helping me, I wasn't scared anymore.”

Transition and follow-up: A illustrated home care manual on anticoagulation management was provided at discharge. A WeChat-based follow-up group was established for ongoing medication adherence support and activity guidance. At 6-month follow-up, the patient had resumed normal schooling and social activities with no evidence of psychological maladjustment.

### 3.6 Comparison with published cases

A search of the literature reveals that ECMO-supported management of acute PE in pediatric patients with inherited thrombophilia is exceptionally rare. Most published pediatric ECMO case reports focus on respiratory failure from pneumonia or sepsis. A 2025 case report described ECMO management in a child with severe lung abscess complicated by bronchopleural fistula, but the anticoagulation challenge in that context differs substantially from the thrombophilia-plus-thrombolysis scenario presented here (13).

The PEACE consensus represents a recent advance in pediatric ECMO anticoagulation, and this case provides one of the first published descriptions of its application in a patient with concurrent thrombophilia and recent thrombolysis. The successful outcome supports the consensus recommendation that low-dose heparin can be safely employed in high-bleeding-risk pediatric ECMO patients, provided that rigorous monitoring is maintained.

### 3.7 Limitations

This report has several limitations. First, as a single case report, the findings may not be generalizable to all pediatric ECMO patients with thrombophilia. Second, the anticoagulation protocol was tailored to this specific patient's characteristics (age, weight, bleeding risk, prior intracranial hemorrhage) and may require modification for other patients. Third, long-term neurodevelopmental outcomes beyond 6 months were not assessed. Future multicenter studies with larger sample sizes are needed to validate the safety and efficacy of this approach across diverse pediatric populations.

## 4 Conclusion

This case demonstrates that individualized low-dose unfractionated heparin anticoagulation, guided by the 2025 PEACE consensus and supported by meticulous monitoring, can successfully balance the competing risks of thrombosis and hemorrhage in a pediatric patient with protein S deficiency and acute PE undergoing ECMO-supported intervention. The nursing strategies employed—including hemodynamic monitoring, multidimensional bleeding prevention, dynamic nutritional support, bundled infection control, and family-involved psychological care—were integral to the favorable outcome.

Key takeaways for clinical practice include: (1) in high-bleeding-risk pediatric ECMO patients, low-dose heparin (starting at 4 U/kg/h) with lower target ranges (ACT 140–160 s, APTT 50–60 s) may be safely implemented; (2) a closed-loop monitoring and titration protocol is essential for maintaining the therapeutic balance; (3) proactive, multidimensional bleeding prevention—including standardized puncture site care, systematic surveillance, and timely intervention for gastrointestinal bleeding—can reduce major hemorrhage risk; and (4) family-involved psychological support is critical for pediatric patients’ recovery from critical illness.

This case provides a practical reference for clinicians and nurses managing similar rare and complex pediatric critical care scenarios. Further research is warranted to establish standardized protocols for anticoagulation management in pediatric ECMO patients with underlying thrombophilia.

## Data Availability

The original contributions presented in the study are included in the article/Supplementary Material, further inquiries can be directed to the corresponding author.
